# Clinical and molecular stratification of disease risk in medulloblastoma

**DOI:** 10.1054/bjoc.2001.1987

**Published:** 2001-09

**Authors:** R Gilbertson, C Wickramasinghe, R Hernan, V Balaji, D Hunt, D Jones-Wallace, J Crolla, R Perry, J Lunec, A Pearson, D Ellison

**Affiliations:** Dept. Developmental Neurobiology, St Jude Children's Research Hospital, Room 2006G, 332 North Lauderdale St, Memphis, TN, 38105, USA; Cancer Research Unit, The Medical School, University of Newcastle upon Tyne, Framlington Place, Newcastle upon Tyne, NE2 4HH, England; Dept. of Cellular Pathology, Southampton General Hospital, Mail point 02, Southampton, SO16 6YD, England; Department of Biostatistics, St Jude Children's Research Hospital, 332 North Lauderdale St, Memphis, TN, 38105, USA; Wessex Regional Genetics Laboratory, Salisbury District Hospital, Salisbury, SP2 8BJ, England; Department of Neuropathology, Newcastle General Hospital, Westgate Road, Newcastle Upon Tyne, NE4 6BE, England; North of England Children's' Cancer Research Unit, Royal Victoria Infirmary, Sir James Spence Building, Queen Victoria Road, Newcastle Upon Tyne, NE1 4LP, England

**Keywords:** medulloblastoma, prognosis, ErbB2, *MYC*, chromosome 17

## Abstract

The accurate assessment of disease risk among children with medulloblastoma remains a major challenge to the field of paediatric neuro-oncology. In the current study we investigated the capacity of molecular abnormalities to increase the accuracy of disease risk stratification above that afforded by clinical staging alone. 41 primary medulloblastoma tumour samples were analysed for ErbB2 receptor expression using immunohistochemistry, and for aberrations of chromosome 17 and amplification of the *MYC* oncogene using fluorescence in situ hybridisation. The ErbB2 receptor and deletion of 17p were detected in 80% and 49% of tumours, respectively. 17p loss occurred either in isolation (20%), or in association with gain of 17q (29%), compatible with an isochromosome of 17q. Amplification of *MYC* was detected in only 2 tumours. Significant prognostic factors included, ‘metastatic disease’ (*P* = 0.0006), ‘sub-total tumour resection’ (*P* = 0.007), ‘high ErbB2 receptor expression’ (*P* = 0.003) and ‘isolated 17p loss’ (*P* = 0.003). Combined analysis of clinical and molecular factors enabled greater resolution of disease risk than clinical factors alone, identifying a sub-population of patients with particularly favourable disease outcome. These data support the hypothesis that a combination of clinical and molecular factors may afford a more reliable means of assigning disease risk in patients with medulloblastoma, thereby providing a more accurate basis for targeting therapy in children with this disease. © 2001 Cancer Research Campaign http://www.bjcancer.com


*British Journal of Cancer* (2001) **85**(5), 705–712
© 2001 Cancer Research Campaign
doi: 10.1054/ bjoc.2001.1987, available online at http://www.idealibrary.com on http://www.bjcancer.com

### Clinical and molecular stratification of disease risk in medulloblastoma

**R Gilbertson[1,2], C Wickramasinghe[3], R Hernan[1,2], V Balaji[3], D Hunt[4], D Jones-Wallace[4], J Crolla[5], R Perry[6], J Lunec[2], A Pearson[7] and D Ellison[2,3,6]**

[1]Dept. Developmental Neurobiology, Room 2006G, St Jude Children's Research Hospital, 332 North Lauderdale St, Memphis TN 38105, USA; [2]Cancer Research Unit, The Medical School, Framlington Place, University of Newcastle upon Tyne, Newcastle upon Tyne NE2 4HH, England; [3]Dept. of Cellular Pathology, Mail point 02, Southampton General Hospital, Southampton SO16 6YD, England; [4]Department of Biostatistics, St Jude Children's Research Hospital, 332 North Lauderdale St, Memphis TN 38105, USA; [5]Wessex Regional Genetics Laboratory, Salisbury District Hospital, Salisbury SP2 8BJ, England; [6]Department of Neuropathology, Newcastle General Hospital, Westgate Road, Newcastle Upon Tyne NE4 6BE, England; [7]North of England Children's' Cancer Research Unit, Sir James Spence Building, Royal Victoria Infirmary, Queen Victoria Road, Newcastle Upon Tyne NE1 4LP, England

**Summary** The accurate assessment of disease risk among children with medulloblastoma remains a major challenge to the field of paediatric neuro-oncology. In the current study we investigated the capacity of molecular abnormalities to increase the accuracy of disease risk stratification above that afforded by clinical staging alone. 41 primary medulloblastoma tumour samples were analysed for ErbB2 receptor expression using immunohistochemistry, and for aberrations of chromosome 17 and amplification of the *MYC* oncogene using fluorescence in situ hybridisation. The ErbB2 receptor and deletion of 17p were detected in 80% and 49% of tumours, respectively. 17p loss occurred either in isolation (20%), or in association with gain of 17q (29%), compatible with an isochromosome of 17q. Amplification of *MYC* was detected in only 2 tumours. Significant prognostic factors included, 'metastatic disease' ($P = 0.0006$), 'sub-total tumour resection' ($P = 0.007$), 'high ErbB2 receptor expression' ($P = 0.003$) and 'isolated 17p loss' ($P = 0.003$). Combined analysis of clinical and molecular factors enabled greater resolution of disease risk than clinical factors alone, identifying a sub-population of patients with particularly favourable disease outcome. These data support the hypothesis that a combination of clinical and molecular factors may afford a more reliable means of assigning disease risk in patients with medulloblastoma, thereby providing a more accurate basis for targeting therapy in children with this disease. © 2001 Cancer Research Campaign http://www.bjcancer.com

**Keywords**: medulloblastoma; prognosis; ErbB2; *MYC*; chromosome 17

The development and assessment of treatment protocols for children with medulloblastoma, the commonest malignant brain tumour of childhood, has led to the understanding that not all patients share the same disease risk. Indeed, a number of clinical variables have been identified that enable the division of patients into 2 broad risk groups. Patients greater than 3 years of age, with no evidence of metastatic disease at diagnosis, who achieve total or near-total tumour resection are regarded as 'standard' risk. Around 80% of these cases may be expected to achieve cure (Zeltzer et al, 1999; Kortmann et al, 2000). In contrast, younger patients, or those with ≥ 1.5 $cm^2$ of residual disease after surgery and/or evidence of metastasis fare much worse, demonstrating long-term survival rates of equal to or less than 50% (Zeltzer et al, 1999; Kortmann et al, 2000).

The capacity to stratify patients with medulloblastoma on the basis of disease risk has undoubted clinical value, enabling the tailoring of treatment intensity to the level of disease aggression. Unfortunately, although clinical variables permit broad distinctions to be made between patient risk groups, when used alone they have proved an unreliable basis for assigning therapy. For example, a recent joint Children's Cancer Group (CCG) and Pediatric Oncology Group (POG) study (CCG-923/POG-8631) which randomised 'standard' risk medulloblastoma patients to receive reduced (23.4 Gy) versus standard (36 Gy) neuraxis radiotherapy, was closed early after an increased risk of relapse was detected among patients in the reduced treatment arm (Thomas et al, 2000). Furthermore, although a second, non-randomised CCG study (CCG-9892) of 23.4 Gy neuraxis radiotherapy in patients with non-metastatic medulloblastoma aged greater than 3 years achieved a 3-year progression-free survival rate in excess of 90%, the study's authors raised appropriate concern regarding the observed pattern of disease relapse (Packer et al, 1999). Of 14 cases experiencing treatment failure, 12 patients suffered disease recurrence outside the primary site.

Together, these data suggest that while the concept of 'standard' risk medulloblastoma is likely to be real, clinical variables alone are an insufficient means of accurately defining disease risk. Stratification on the basis of both clinical and molecular prognostic markers, potentially provides a more accurate means of identifying patients for trials of treatment reduction. Therefore, in the current study we have investigated the prognostic significance of 3 molecular markers (*ErbB2* oncogene protein expression, abnormality of chromosome 17 and *MYC* oncogene amplification), previously implicated in promoting aggressive biological behaviour in medulloblastoma (Badiali et al, 1991; Cogen, 1991; Batra et al, 1995; Gilbertson et al, 1997a, 1997b; Herms et al, 1997; Scheurlen et al, 1998; Herms et al, 2000). In particular, we have assessed the capacity of these molecular variables to provide

*Received 26 January 2001*
*Revised 30 April 2001*
*Accepted 15 May 2001*

*Correspondence to:* R Gilbertson prognostic information additional to that afforded by contemporary clinical staging.

### MATERIAL AND METHODS

### Patients

The study population included 41, consecutively treated patients, aged ≥3 years, with newly diagnosed medulloblastoma, treated at Newcastle and Southampton General Hospitals, England, between 1984 and 1997. The clinical details of each patient are summarised in Table 1. 12 of the 41 cases were drawn from a cohort of 70 patients whose tumours were previously investigated by us for ErbB2 expression using immunohistochemical (IHC) analysis (Gilbertson et al, 1997a). These patients were included because they fulfilled the eligibility criteria for the study and had not previously been investigated for chromosome 17 abnormality or *MYC* oncogene amplification. The histological diagnosis of medulloblastoma was confirmed according to WHO criteria (Giangaspero et al, 2000). 19 cases fulfilled the diagnostic criteria for desmoplastic medulloblastoma. No cases of atypical teratoid rhabdoid tumour were identified within the study population. Metastatic disease stage and extent of surgical resection were assessed using a combination of pre- and postoperative CT and MRI brain and spinal imaging. CSF sampling for the assessment of M1 status is not routinely conducted in the United Kingdom, therefore patients were classified as either <M2 or ≥M2. 21 patients received standard radiotherapy (55 Gy to the primary tumour and 35 Gy to the neuraxis) alone following surgery. In addition to standard radiotherapy, a further 19 patients all received and completed pre-irradiation chemotherapy randomised by the 2nd or 3rd International Society of Pediatric Oncology (SIOP) trials (Bailey et al, 1995). The median follow up time for the 22 patients alive at last follow up was 4.5 years (range 0.2–12 years).

### Immunohistochemical (IHC) analysis of ErbB2 receptor expression

Protocols for the fixation and histological processing of tumour samples were identical between the 2 study centres. Analysis of ErbB2 expression was conducted in 5 μm formalin-fixed paraffin wax-embedded tumour sections using the anti-ErbB2 monoclonal antibody NCL-CB11 (Novocastra, UK) as described in detail elsewhere (Gilbertson et al, 1997a). In our previous studies a score of greater than 50% ErbB2 immunopositive tumour cells was associated with a worse clinical outcome (Gilbertson et al, 1997a). Therefore, in the current study, tumours were scored as either ‘high’ ErbB2 expressing (≥50% immunopositive tumour cells) or ‘low’ ErbB2 expressing (<50% immunopositive tumour cells) by 2 independent observers (RG and RH). No discrepancies in staining score were encountered.

### Fluorescence in situ hybridisation (FISH)

Fluorescence in situ hybridisation (FISH) was employed to identify both abnormalities of chromosome 17 and *MYC* oncogene amplification in material derived from each of the tumour blocks employed in the analysis of ErbB2 receptor expression. The method is described in detail elsewhere (Nicholson et al, 2000). For the analysis of imbalance on the short and long arms of chromosome 17, cytospin preparations of nuclei from sections of formalin-fixed, wax-embedded tumour or tonsillar tissue (normal controls) were probed with a digoxigenin-labelled cosmid probe to the subtelomeric region of 17p (2111b1) or 17q (B37c1) in combination with a 17 centromeric biotin-labelled plasmid probe (D17Z1). A digoxigenin-labelled BAC probe (968N11) encompassing the *MYC* gene was combined with a biotin-labelled plasmid probe (D8Z1) to the alpha-satellite repeat region of chromosome 8 for analysis of *MYC* amplification. FISH signals for each case were assessed in 200 non-overlapping nuclei as described previously (Nicholson et al, 2000).

### Statistical analysis

Relationships between molecular and clinical markers were assessed using Fisher’s and Chi-square exact tests. Survival was measured from the date of initial diagnosis of medulloblastoma to the date of death or last contact. Survival distributions were assessed using Kaplan–Meier curves and their standard errors calculated using the method described by Peto et al (1977). The relatively small number of study patients, further reduced within levels of each prognostic factor, required calculating and reporting exact $P$ values. The exact $P$ value allows analysis of small, sparse or unbalanced data because it is not based on large sample assumptions (Mantel, 1966). Exact tests were accomplished using the software package StatXact (Cytel Software Corp, Cambridge, MA).

### RESULTS

### ErbB2 receptor expression

Sufficient tumour material was available for the analysis of ErbB2 receptor expression in all but one (case 5) of the 41 patients. 28% ($n$ = 9/32) of positive cases were designated as ‘high’ expressing tumours, demonstrating intense mixed cytoplasmic and membrane ErbB2 immunoreactivity in ≥50% of tumour cells (Table 1 and Figure 1).

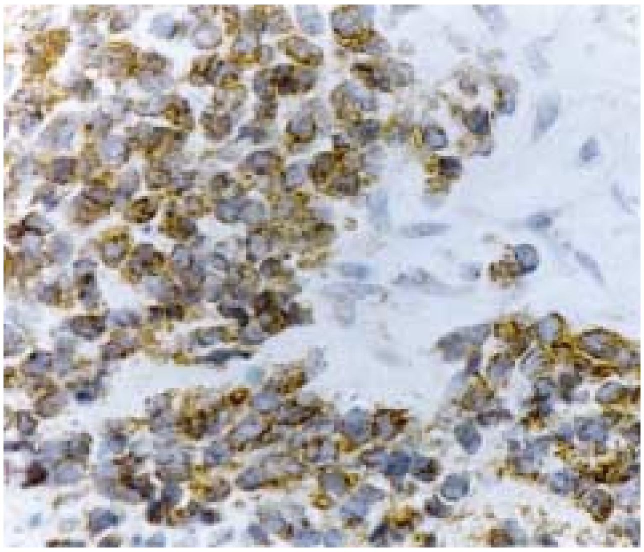

**Figure 1** Immunohistochemical analysis of ErbB2 receptor expression in medulloblastoma. Photomicrograph of a sample taken from the study population demonstrating receptor expression in >50% of tumour cells (mag × 200)

 © 2001 Cancer Research Campaign

BJOC 01-1987 705-712 20/8/01 3:49 pm Page 707

### FISH analysis of chromosome 17 and *MYC* oncogene amplification

FISH analysis of chromosome 17 was conducted in all 41 study patients (Table 1). Loss of 17p was detected in 20 (49%) tumours. In 12 of these, additional gain of 17q was also observed, generating a pattern of imbalance compatible with an isochromosome of 17q (i(17q), Figure 2A). Loss of 17p with a normal 17q (termed hereafter 'isolated 17p loss'), was observed in the remaining 8 cases (Figure 2B). No abnormality of chromosome 17p could be detected in 21 (51%) study cases (Figure 2C).

Availability of tumour material restricted FISH analysis of *MYC* oncogene copy number to 34 cases (Table 1). Gene amplification was observed in only 6% (*n* = 2/34, cases 7 and 31, Figure 2D). Both cases demonstrated numerous copies of the *MYC* gene (Figure 2D).

### Relationship between molecular, histopathological and clinical variables

A significant, positive relationship was observed between 'high' expression of the ErbB2 receptor and 'isolated 17p loss', with 'normal' or 'i(17q)' compatible FISH patterns occurring more frequently in tumours with 'low' ErbB2 expression ($P = 0.01$). In addition, a positive trend was observed between the presence of metastases at diagnosis and 'high' ErbB2 tumour expression, however this did not reach significance ($P = 0.08$). No other significant relationship was observed between any of the other clinicopathological variables. Importantly, we observed no significant relationship between patient age and any molecular abnormality. Specifically, no significant clustering either including or excluding chromosome 17 abnormality, high ErbB2 expression or *MYC* amplification, was seen among the 6 patients in the study population (36–41) aged between 20 and 30 years.

**Table 1** Summary of the clinical and molecular characteristics of the study population

| Study no. | Sex | Age (y) | Phenotype | Metastasis | Surgery | Therapy | Follow up (months) | Status | Chr. 17 | ErbB2 | *MYC* |
|---|---|---|---|---|---|---|---|---|---|---|---|
| 1 | F | 18 | C | Y | P | R | 18 | DOD | i17q | L | – |
| 2 | M | 4.4 | C | N | T | CR | 144 | A | n | L | – |
| 3 | M | 12 | C | N | T | CR | 140 | A | n | L | – |
| 4 | F | 5 | C | Y | P | R | 6 | DOD | –17p | H | – |
| 5 | F | 5 | D | N | T | R | 137 | A | n | ND | – |
| 6 | M | 9.3 | D | Y | P | CR | 10 | DOD | –17p | H | – |
| 7 | M | 12.7 | D | N | T | CR | 109 | A | n | L | + |
| 8 | M | 19.6 | D | N | P | R | 54 | DOD | n | L | – |
| 9 | F | 18.5 | C | N | P | CR | 53 | DOD | –17p | H | ND |
| 10 | M | 14.3 | C | N | P | CR | 121 | A | i17q | L | – |
| 11 | M | 8.5 | C | N | P | CR | 40 | DOD | i17q | L | – |
| 12 | M | 6.3 | C | Y | T | CR | 17 | DOD | i17q | H | – |
| 13 | F | 3.6 | D | N | T | R | 104 | A | n | L | – |
| 14 | M | 9.8 | C | Y | P | CR | 20 | DOD | i17q | L | – |
| 15 | M | 5.1 | D | N | P | CR | 96 | A | i17q | L | – |
| 16 | M | 6.5 | C | N | P | CR | 61 | DOD | n | L | ND |
| 17 | M | 3.3 | C | N | T | R | 90 | A | i17q | L | ND |
| 18 | M | 5.2 | D | N | P | N | 1 | DOD | i17q | H | – |
| 19 | M | 4.7 | C | N | T | CR | 45 | A | i17q | L | – |
| 20 | F | 19.8 | C | N | P | R | 73 | A | n | L | – |
| 21 | M | 20 | C | Y | P | R | 20 | DOD | n | L | – |
| 22 | F | 4.75 | C | Y | T | CR | 13 | DOD | –17p | L | – |
| 23 | F | 10 | C | N | T | CR | 23 | DOD | –17p | L | – |
| 24 | M | 7.25 | D | N | T | R | 57 | A | n | L | – |
| 25 | M | 6.6 | C | N | T | CR | 45 | A | n | L | – |
| 26 | M | 7.5 | D | N | T | R | 50 | A | n | L | – |
| 27 | M | 6.5 | D | N | T | CR | 7 | A | n | L | – |
| 28 | M | 4.3 | C | Y | T | R | 6 | A | n | H | – |
| 29 | F | 16.7 | D | N | T | R | 11 | A | n | L | ND |
| 30 | M | 5.3 | D | Y | P | CR | 42 | A | –17p | H | ND |
| 31 | F | 4.2 | C | N | P | CR | 5 | A | n | H | + |
| 32 | M | 11.2 | D | N | P | R | 47 | A | i17q | L | – |
| 33 | M | 6.7 | C | N | P | R | 2 | A | n | L | – |
| 34 | F | 5.9 | D | N | P | CR | 48 | A | i17q | L | ND |
| 35 | M | 4 | D | N | P | R | 40 | A | n | L | ND |
| 36 | M | 24.7 | C | Y | P | R | 18 | DOD | i17q | L | – |
| 37 | M | 30.2 | D | N | P | R | 29 | DOD | –17p | H | – |
| 38 | M | 30.2 | C | N | P | R | 40 | DOD | –17p | L | – |
| 39 | M | 25.6 | D | *ND* | T | R | 77 | DOD | n | L | – |
| 40 | F | 22.7 | D | N | T | R | 111 | A | n | L | – |
| 41 | M | 27.9 | D | Y | P | R | 67 | DOD | n | L | – |

C, classic medulloblastoma; D, desmoplastic medulloblastoma; Y, M2+ disease; N, <M2 disease; P, partial resection; T, total resection; CR, chemotherapy and radiotherapy; R, radiotherapy only; DOD, dead of disease; A, alive; n, 'normal'; i17q, 'isochromosome 17q'; –17p, 'isolated 17p loss' compatible FISH pattern; L, 'Low' (<50% ErbB2 positive tumour cells); H, 'High; (≥50% ErbB2 positive tumour cells); ND, not done; –, not amplified; +, amplified.

*© 2001 Cancer Research Campaign* 

BJOC 01-1987 705-712 20/8/01 3:50 pm Page 708

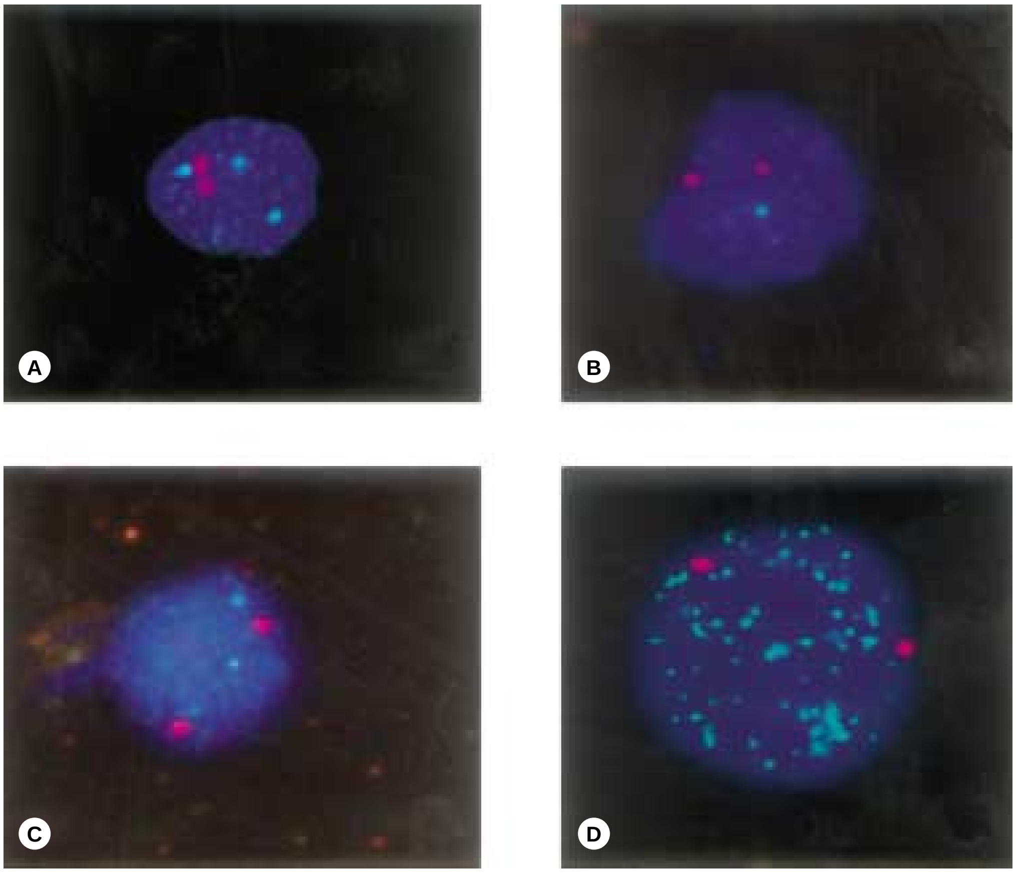

**Figure 2** Fluorescence in situ hybridization (FISH) analysis of medulloblastoma samples taken from patients in the study population. (**A**) 'i(17q)' compatible pattern, showing three 17q subtelomeric (green) and two 17 centromeric (red) probe signals. (**B**) 'Isolated 17p loss' compatible pattern, showing a single 17p subtelomeric (green) and two 17 centromeric (red) probe signals. (**C**) 'Normal' chromosome 17 compatible pattern showing two 17p subtelomeric (green) and two 17 centromeric (red) probe signals. (**D**) FISH analysis of case 7 demonstrating multiple *MYC* oncogene probe signals (green) compatible with gene amplification

In contrast to the report of Scheurlen and colleagues (Scheurlen et al, 1998), we did observe 17p deletion within desmoplastic tumours (Table 1). The low frequency of *MYC* oncogene amplification observed in the current study precluded any analysis of its relationship to other clinicopathological variables including survival.

### Univariate survival analysis

Among the clinical variables analysed, only the degree of tumour resection ($P = 0.007$) and metastatic disease stage at diagnosis ($P = 0.0006$) related significantly to overall survival, confirming their well established prognostic significance in medulloblastoma (Zeltzer et al, 1999). In contrast, neither the type of adjuvant therapy received ('chemotherapy and radiotherapy' vs. 'radiotherapy alone', $P = 0.8$), histological subtype ($P = 0.06$) or age at diagnosis (≥3–<10 years vs. ≥10 years, $P = 0.07$), appeared to influence clinical outcome. However, the numbers involved in the current study are too small to dismiss these variables as insignificant prognostic markets. The absence of a significant relationship between patient age and survival is compatible with inclusion in the study of only patients aged ≥3 years and a lack of any observed relationship between patient age and other clinical or molecular risk factors.

Expression of the ErbB2 receptor and chromosome 17 subtype were both significantly related to survival (Figures 3A, B). Of 9 patients identified with 'high' ErbB2 expressing tumours, 7 (78%) have died from their disease, all within 5 years of diagnosis. This is in contrast to only 12 deaths among 31 patients (39%) with 'low' expressing tumours (Figure 3A, $P = 0.003$). It is important to note that only 3 of the 12 patients previously analysed by us for ErbB2 expression had 'high' expressing tumours, and their inclusion did not bias the statistical analysis. A significant survival difference was also detected between at least 2 of the 3 chromosome 17 subgroups (Figure 3B, $P = 0.013$). Disease outcome was noted to be especially poor in those patients with 'isolated 17p loss'. Of the 8 patients within this group, 7 (88%) have died from their disease, 5 within 3 years of diagnosis (Figure 3B). Pairwise analysis of patients with 'isolated 17p loss' and 'normal' chromosome 17 FISH pattern disease, confirmed a significant difference in survival between these 2 groups ($P = 0.003$). It was not possible to determine whether the survival of patients whose tumours were classified as 'i(17q)' was significantly different from the other 2 subgroups. Indeed, while the short-term disease course of these patients was similar to that of 'isolated 17p loss' cases (Figure 3B), long-term clinical outcome was closer to that of patients whose tumours demonstrated a 'normal' chromosome 17 FISH pattern, with 50% ($n = 6/12$) of 'i(17q)' cases remaining alive at time of analysis with a median follow 69 months (range 45–121

© 2001 Cancer Research Campaign

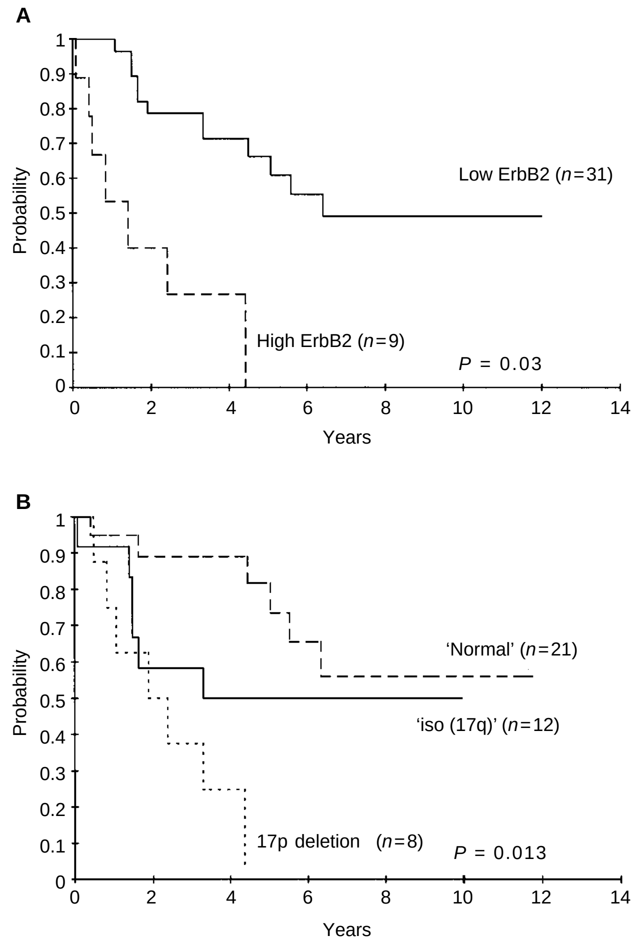

**Figure 3** Kaplan–Meier survival curves demonstrating the influence of molecular prognostic markers on the overall survival rate of patients in the study population. (**A**) ErbB2 receptor expression and survival. (**B**) Chromosome 17 aberration detected by FISH and survival months). These data support the hypothesis that 'isolated 17p loss' and 'high' ErbB2 receptor expression are biological markers of poor clinical outcome in patients with medulloblastoma.

Close inspection of the patient population revealed 2 cases each with 'normal' (cases 28 and 31) or 'i(17q)' (cases 12 and 18) FISH patterns but 'high' expression of ErbB2, and 3 cases with 'isolated 17p loss', 'low' ErbB2 expressing tumours (cases 22, 23, 38). Therefore, analysis of a single molecular marker may result in erroneous underestimation of disease risk for patients whose tumour harbours only one abnormality. To study this further, patients were divided into 2 molecular risk groups. 'High' molecular risk patients were designated as those with 'high' ErbB2 receptor expression and/or 'isolated 17p loss', while 'low' molecular risk patients were designated as patients whose tumours demonstrated neither aberration. Of 12 patients classed as molecular 'high' risk, 83% ($n$ = 10/12) have died from their disease, 8 within 3 years of diagnosis. In contrast, only 32% ($n$ = 9/28) of 'low' molecular risk patients have succumbed to their disease. This risk stratification enabled a greater level of resolution between patients with 'high' and 'low' risk disease than any clinical or molecular marker analysed individually (Figure 4A, $P$ = 0.0001).

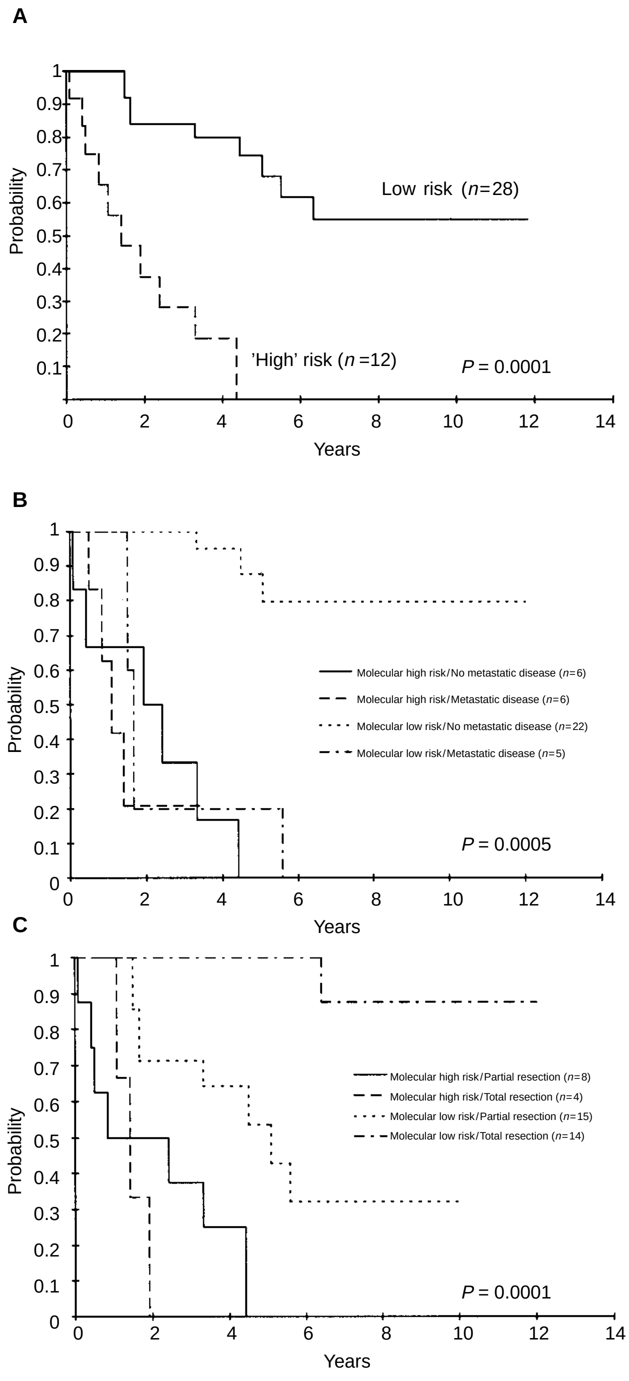

**Figure 4** Kaplan–Meier survival curves demonstrating the prognostic significance of molecular risk group alone (**A**), and in combination with clinical factors (**B**) metastatic stage (< M2 vs. ≥ M2 disease) and (**C**) extent of surgical resection ('total' vs. 'partial')

### Survival analysis following clinical risk stratification

The principal aim of this study was to establish whether molecular abnormalities provide prognostic information for patients with medulloblastoma additional to that afforded by clinical risk stratification. Therefore, we repeated the survival analysis of molecular risk groups, but first stratified patients for either metastatic disease stage or the degree of surgical resection.

*© 2001 Cancer Research Campaign* 

BJOC 01-1987 705-712 20/8/01 3:50 pm Page 710

First, patients were grouped into 1 of 4 catagories: 'non-metastatic, high molecular risk' (*n* = 6), 'non-metastatic, low molecular risk' (*n* = 22), 'metastatic, high molecular risk' (*n* = 6) or 'metastatic, low molecular risk' (*n* = 5). Patients with 'non-metastatic, low molecular risk' disease (*n* = 22) had a significantly better survival than any other risk group, with only 3 of these cases (13%) dying from disease and median follow-up time in the 20 surviving cases exceeding 5 years (Figure 4B, *P* = 0.0005). Of particular note, 5 of 6 patients presenting with 'non-metastatic, high molecular risk' tumours, died from their disease within 5 years of diagnosis, indicating the ability of molecular risk stratification to refine disease risk attributed by clinical variables alone.

The value of combining clinical and molecular risk staging was illustrated further following stratification according to degree of surgical resection (Figure 4C). Patients were again grouped into 1 of 4 categories: 'partial resection, high molecular risk' (*n* = 8), 'partial resection, low molecular risk' (*n* = 15), 'total resection, high molecular risk' (*n* = 4) or 'total resection, low molecular risk' (*n* = 14). Of the 14 patients undergoing total resection of low molecular risk tumours, only 1 patient (7%) has died from disease. Furthermore, the median survival time of the remaining 13 cases has exceeded 7 years (median 90 months, range 11–144), with no evidence of disease relapse at time of follow up (Figure 4C). Although patient numbers in these risk groups are small, exact log-rank analysis again allowed a cohort of particularly good-risk patients to be clearly resolved from the remaining 3 categories, including those patients with 'totally resected, high molecular risk' tumours (Figure 4C, *P* = 0.0001), indicating the additional prognostic information afforded by molecular risk stratification.

### DISCUSSION

The treatment of medulloblastoma is at an impasse. Around 80% of patients currently classified by clinical criteria as 'standard' risk may achieve long-term survival (Zeltzer et al, 1999). However, the majority will suffer major neurological and endocrine side effects secondary to neuraxis radiotherapy (Schwartz, 1999). Attempts to reduce the intensity of radiation treatment in these patients have proved difficult due to unacceptable relapse rates (Packer et al, 1999; Thomas et al, 2000). Furthermore, treatment options for patients with macroscopic metastases at diagnosis or for those whose disease fails to respond to conventional therapy are extremely limited, with 40% and under 5% of such cases respectively achieving cure (Zeltzer et al, 1999; Kortmann et al, 2000).

These observations provide 2 clear challenges for the future management of children with medulloblastoma. First, additional markers other than clinical factors are required if we are to identify those patients who can be cured with reduced therapy. Second, it is imperative that novel therapeutic approaches are developed to improve the survival of patients with more aggressive disease. It is likely that these challenges will be met only by improved knowledge of the biology of medulloblastoma. To this end, we investigated the frequency and prognostic significance of ErbB2 receptor expression, chromosome 17 abnormalities and *MYC* amplification in the largest combined analysis of these molecular abnormalities in medulloblastoma to be conducted to date. For the first time, we provide evidence that molecular factors may allow increased accuracy over clinical stratification alone in the assignment of medulloblastoma disease risk.

In the current study, amplification of the *MYC* oncogene was identified in only 6% of analysed tumours (*n* = 2/34, cases 9 and 34). This incidence is identical to the overall *MYC* amplification rate of 6% (*n* = 9/132 total tumours analysed) reported by the majority of published studies (Badiali et al, 1991; Batra et al, 1995; Herms et al, 2000). One group has identified *MYC* amplification in 17% (5/29) of medulloblastomas. However, this increased frequency is likely to reflect the nature of their study material which included primary tumours from clinically high-risk patients, including patients less than 3 years of age and samples of CSF metastases (Scheurlen et al, 1998). Most published studies have reported an adverse effect of *MYC* amplification on clinical outcome (Badiali et al, 1991; Batra et al, 1995). Although the small number of *MYC*-amplified cases detected in the current series precluded formal survival analysis, it is noteworthy that one patient (7) remains alive and disease-free more than 9 years after diagnosis.

Deletions involving the short arm of chromosome 17 represent the most frequent genetic abnormality in medulloblastoma, occurring in 40–50% of primary tumours (Cogen, 1991; Batra et al, 1995; Emadian et al, 1996; Biegel et al, 1997; Scheurlen et al, 1997, 1998; Nicholson et al, 2000). Susceptibility to 17p deletion appears to result from the presence of at least 4 breakpoint cluster regions distributed within the centromeric, Charcot–Marie–Tooth and Smith–Magenis syndrome loci of 17p (Scheurlen et al, 1997), and may occur in the absence of other gross abnormalities of 17, or more frequently as a component of an i(17q) (Scheurlen et al, 1997; Nicholson et al, 2000). In keeping with these studies, we identified loss of the telomeric region of 17p in 49% (*n* = 20/41) of tumours, with 60% (*n* = 12/20) of these cases demonstrating concurrent gain of 17q compatible with the formation of an i(17q).

At present, the clinical and molecular significance of 17p deletion in medulloblastoma remains unclear. Although a number of studies in the literature have reported a significantly worse prognosis for patients whose tumours harbour deletions of 17p (Cogen, 1991; Batra et al, 1995; Scheurlen et al, 1997) this has not been a universal finding (Emadian et al, 1996; Biegel et al, 1997). However, these studies have involved only small numbers of cases or employed a variety of techniques with different sensitivities to analyse 17p loss, thereby rendering them difficult to interpret. Very recently, data was presented suggesting that the *hypermethylated in cancer-l* gene (*HIC-1*) located at 17p 13.3 may represent an important gene deleted by such abnormalities, thereby acting as a potential tumour suppressor for medulloblastoma (Eberhart et al, 1999). In the current study, we employed a single, sensitive technique to detect aberration of chromosome 17 in a relatively large, well characterised series of patients and identified a significantly worse clinical outcome for cases whose tumours had 'isolated 17p loss' compared to a 'normal' chromosome 17 FISH pattern (Figure 3B). Patient numbers were too small in our analysis to allow statistical resolution of the survival of patients with an 'i(17q)' compatible pattern from those with 'isolated 17p loss'. However, the current study does provide preliminary evidence that these 2 may represent clinically and biologically distinct tumour subtypes. First, compared to the catastrophic disease course observed among 'isolated 17p loss' cases, the 5-year survival rate of patients whose tumours displayed an 'i(17q)' compatible FISH pattern was 50%, with a median disease-free follow-up time in surviving patients of 69 months (range 45–121 months) (Figure 3B). Second, in our analysis of the relationship between ErbB2 expression and chromosome 17 abnormality, while the majority of chromosome 17 'normal' (*n* = 18/21) and i(17q) (*n* = 10/12) compatible tumours demonstrated 'low' receptor levels, 5 of 8

 © 2001 Cancer Research Campaign

BJOC 01-1987 705-712 20/8/01 3:50 pm Page 711

tumours with ‘isolated 17p loss’ exhibited ‘high’ ErbB2 expression ($P$ = 0.01). Further analysis is required to establish whether ErbB2 overexpression and ‘isolated 17p loss’ are functionally related or represent independent markers of aggressive disease.

The ErbB2 receptor plays a central role in the signalling network formed by the 4 members of the ErbB tyrosine kinase receptor family and their cognate ligands (Olayioye et al, 2000). Operating as the preferred dimer partner of the 3 other family members, this receptor mediates the transduction of potent, highly transforming cellular signals (Olayioye et al, 2000). Considerable evidence exists in the literature implicating the ErbB2 receptor in the pathogenesis of human cancer including medulloblastoma (Gilbertson et al, 1997a, 1997b; Herms et al, 1997; Olayioye et al, 2000). We initially reported a significant relationship between elevated tumour cell proliferation (Gilbertson et al, 1997b) and reduced long-term survival (Gilbertson et al, 1997a) in patients with medulloblastoma who expressed the ErbB2 receptor in ≥ 50% of tumour cells. These data are supported by the results of the current study in which 5-year overall survival rates of 66 ± 11% and 0% were observed for patients with ‘low’ and ‘high’ ErbB2 expressing tumours, respectively (Figure 3A, $P$ = 0.003).

The principal aim of the current study was to investigate the hypothesis that the combined assessment of clinical and molecular prognostic markers may allow increased accuracy of disease-risk stratification for patients with medulloblastoma. Data from the current study strongly support this hypothesis. First, although the ‘extent of surgical resection’, ‘metastatic stage’, ‘level of ErbB2 expression’ and ‘isolated 17p loss’ were all powerful prognostic indicators, no significant relationship could be identified between clinical and molecular factors. This suggests that patients identified as ‘high’ or ‘low’ risk disease by clinical stratification do not completely overlap with those stratified by molecular subgroup analyses. Second, the requirement for multiple rather than single molecular factor analysis was supported by the observation in 7 (17%) tumours of either ‘high’ expression of ErbB2 or ‘isolated 17p loss’ but not both molecular aberrations. Finally, combined clinical and molecular factor analysis enabled the resolution of a subset of patients with a particularly favourable disease outcome (Figure 4B, C). For example, only 3 of 22 patients with non-metastatic ‘low’ molecular risk disease have died from their disease with a median survival of 65 months in surviving patients (range 2–144 months). This is in contrast to 5 deaths among 6 cases with non-metastatic, ‘high’ molecular risk disease, the 1 surviving patient having follow up of only 5 months. Similar results were also achieved in the analysis of surgical resection and disease risk (Figure 4C). Of particular note, all patients in the study ($n$ = 12) undergoing total resection of non-metastatic, ‘low molecular risk’ tumours are alive and disease free with a median follow up of 73 months. Only one other case (23) in the study population achieved total resection of a non-metastatic tumour. This patient had ‘high molecular risk’ disease and died within 2 years of diagnosis.

We are currently seeking to confirm these results in a larger prospective study of patients with medulloblastoma. It is hoped that combined analysis of clinical and molecular risk stratification may significantly improve disease management efficiency, and in particular allow more accurate identification of patients who may be cured with reduced therapy. In this regard it will also be important in future studies to analyse the interaction between molecular genetic abnormalities and treatment response. Neuraxis radiation therapy is routinely employed in medulloblastoma because of the high propensity of this tumour to disseminate throughout the CNS. Although only a non-significant trend between high ErbB2 expression and stage ≥M2 was observed in the current study, we have previously reported a significant relationship between the presence of metastatic disease at diagnosis and high ErbB2 immunoreactivity (Gilbertson et al, 1998). Therefore, tumours expressing low levels of this receptor may indeed exhibit a lower metastatic potential and represent a subgroup in which reduced dose neuraxis radiotherapy may be rationally applied.

Finally, molecular aberrations important in maintaining an aggressive tumour phenotype may also represent potential targets for novel therapeutic approaches. In this regard, the ErbB2 receptor has received considerable attention following the successful development and use of the anti-ErbB2 therapeutic monoclonal antibody Herceptin, in the management of women with high ErbB2-expressing metastatic breast cancer (Cobleigh et al, 1999). Recently we demonstrated that the ErbB2 receptor can be targeted to achieve growth inhibitory effects against medulloblastoma cells in vitro (Gilbertson et al, 1999). Further studies are underway to investigate whether this may indeed represent a potential new therapeutic approach for childhood medulloblastoma.

### ACKNOWLEDGEMENTS

We thank Dr Rocchi, Cytogenetics Unit, Sezione di Genetica, University of Bari, Italy for his generous gift of DNA probes and Mr William MacMeekin, Newcastle General Hospital, Miss Sarah Beal, Wessex Regional Genetics Laboratory, and Mrs Jean Buontempo, Southampton General Hospital, for their excellent technical assistance. This work was supported in part by the United Kingdom Medical Research Council, the Wessex Cancer Trust, the Wolfson Foundation, Cancer Center (CORE) grant CA 21765 and by the American Lebanese Syrian Associated Charities (ALSAC).

### REFERENCES

Badiali M, Pession A, Basso G, Andreini L, Rigobello L, Galassi E and Giangaspero F (1991) N-myc and c-myc oncogenes amplification in medulloblastomas. Evidence of particularly aggressive behavior of a tumour with c-myc amplification. *Tumouri* **77**: 118–121

Bailey CC, Gnekow A, Wellek S, Jones M, Round C, Brown J, Phillips A and Neidhardt MK (1995) Prospective randomised trial of chemotherapy given before raidotherapy in childhood medulloblastoma. International Society of Paediatric Oncology (SIOP) and the German Society of Paediatric Oncology (GPO): SIOP II. *Med Ped Oncol* **25**: 166–178

Batra SK, McLendon RE, Koo JS, Castelino-Prabhu S, Fuchs HE, Krischer JP, Friedman HS, Bigner DD and Bigner SH (1995) Prognostic implications of chromosome 17p deletions in human medulloblastoma. *J Neuro-oncol* **24**: 39–45

Biegel JA, Janss AJ, Raffel C, Sutton L, Rorke LB, Harper JM and Phillips PC (1997) Prognostic significance of chromosome 17p deletions in childhood primitive neuroectodermal tumours (medulloblastomas) of the central nervous system. *Clin Cancer Res* **3**: 473–478

Cobleigh MA, Vogel CL, Tripathy D, Robert NJ, Scholl S, Fehrenbacher L, Wolter JM, Paton V, Shak S, Lieberman G and Slamon DJ (1999) Multinational study of the efficacy and safety of humanized anti-HER2 monoclonal antibody in women who have HER2-overexpressing metastatic breast cancer that has progressed after chemotherapy for metastatic disease. *J Clin Oncol* **17**: 2639–2648

Cogen PH (1991) Prognostic significance of molecular genetic markers in childhood brain tumours. *Pediatr Neurosurg* **92**: 245–250

Eberhart CG, Corn P, Campbell S, Burger PC, Baylin SB and Cohen KJ (1999) *HIC-1 hypermethylation in medulloblastoma*. Presented at the International Society of Pediatric Neuro-oncology, San Francisco June 10–14

© 2001 Cancer Research Campaign 

Emadian SM, McDonald JD, Gerken SC and Fults D (1996) Correlation of chromosome 17p loss with clinical outcome in medulloblastoma. *Clin Cancer Res* **2**: 1559–1564

Giangaspero F, Bigner SH, Kleihues P, Pietsch T and Trojanowski J (2000) Medulloblastoma. In: *World Health Organisation classification of tumours pathology & genetics tumours of the central nervous system* (Kleihues P, Cavenee WK, eds). IARC press: Lyon

Gilbertson RJ, Perry RH, Kelly PJ, Pearson AD and Lunec J (1997a) Prognostic significance of HER2 and HER4 coexpression in childhood medulloblastoma. *Cancer Res* **57**: 3272–3280

Gilbertson RJ, Jaros E, Perry RH, Kelly PJ, Lunec J and Pearson AD (1997b) Mitotic percentage index: A new prognostic factor for childhood medulloblastoma. *Eur J Cancer* **33**: 609–615

Gilbertson RJ, Clifford SC, MacMeekin W, Mcckin W, Wright C, Perry RH, Kelly P, Pearson AD and Lunec J (1998) Expression of the ErbB-Neuregulin signaling network during human cerebellar development: Implications for the biology of medulloblastoma. *Cancer Res* **58**: 3932–3941

Gilbertson RJ, Hernan R, Pearson ADJ, Pietsch T and Lunec J (1999) The ErbB2 receptor represents a potential target for novel therapies in childhood medulloblastoma. *Clin Cancer Res* **5** (Suppl 11): 3737

Herms JW, Behnke J and Bergmann M (1997) Potential prognostic value of c-erbB-2 expression in medulloblastoma in very young children. *J Ped Hematol Oncol* **19**: 510–515

Herms J, Neidt I, Luscher B, Sommer A, Schurmann P, Schroder T, Bergmann M, Wilken B, Probst-Cousin S, Hernaiz-Driever P, Behnke J, Hanefeld F, Pietsch T and Kretzschmar HA (2000) C-MYC expression in medulloblastoma and its prognostic value. *Int J Cancer* **89**: 395–402

Kortmann RD, Kuhl J, Timmermann B, Mittler U, Urban C, Budach V, Richter E, Willich N, Flentje M, Berthold F, Slavc I, Wolff J, Meisner C, Wiestler O, Sorensen N, Warmuth-Metz M and Bamberg M (2000) Postoperative neoadjuvant chemotherapy before radiotherapy as compared to immediate radiotherapy followed by maintenance chemotherapy in the treatment of medulloblastoma in childhood: Results of the German prospective randomised trial HIT '91. *Int J Radiat Oncol Biol Phys* **46**: 269–279

Mantel N (1966) Evaluation of survival data and two new rank order statistics arising in its consideration. *Cancer Chemother Rep* **50**: 163–170

Nicholson JC, Wickramasinghe CL, Ross FM, Crolla J and Ellison DW (2000) Imbalances of chromosome 17 in medulloblastomas determined by comparative genomic hybridisation and fluorescence in situ hybridisation. *J Clin Path: Mol Path* **53**: 313–319

Olayioye MA, Neve RM, Lane HA and Hynes N (2000) The ErbB signaling network: receptor heterodimerisation in development and cancer. *EMBO* **19**: 3159–3167

Packer RJ, Goldwein J, Nicholson HS, Vezina LG, Allen JC, Ris MD, Muraszko K, Rorke LB, Wara WM, Cohen BH and Boyett JM (1999) Treatment of children with medulloblastoma with reduced-dose craniospinal radiation therapy and adjuvant chemotherapy: A Children's Cancer Study Group study. *J Clin Oncol* **17**: 2127–2136

Peto R, Pike MC, Armitage P, Breslow NE, Cox DR, Howard SV, Mantel N, McPherson K, Peto J and Smith PG (1977) Design and analysis of randomized clinical trials requiring prolonged observation of each patient, II: analysis and examples. *Br J Cancer* **35**: 1–39

Scheurlen WG, Seranski P, Mincheva A, Kuhl J, Sorensen N, Krauss J, Lichter P, Poustka A and Wilgenbus KK (1997) High-resolution mapping of chromosome arm 17p in childhood primitive neuroectodermal tumours reveals a common chromosomal disruption within the Smith-Magenis region, an unstable chromosome band 17p 11.2. *Gene Chrom Cancer* **18**: 50–58

Scheurlen WG, Schwabe GC, Joos S, Mollenhauer J, Sorensen N and Kuhl J (1998) Molecular analysis of childhood primitive neuroectodermal tumours defines markers associated with poor clinical outcome. *J Clin Oncol* **16**: 2478–2485

Schwartz CL (1999) Long-term survivors of childhood cancer: The late effects of therapy. *Oncologist* **4**: 45–54

Thomas PR, Deutsch M, Kepner JL, Boyett JM, Krischer J, Aronin P, Albright L, Allen JC, Packer RJ, Linggood R, Mulhern R, Stehbens JA, Langston J, Stanley P, Duffner P, Rorke L, Cherlow J, Friedman HS, Finlay JL, Vietti TJ and Kun LE (2000) Low-stage medulloblastoma: Final analysis of trial comparing standard-dose with reduced dose neuraxis irradiation. *J Clin Oncol* **18**: 3004–3011

Zeltzer PM, Boyett JM, Finlay JL, Albright AL, Rorke LB, Milstein JM, Allen JC, Stevens KR, Stanley P, Li H, Wisoff JH, Geyer JR, McGuire-Cullen P, Stehbens JA, Shurin SB and Packer RJ (1999) Metastasis stage, adjuvant treatment, and residual tumour are prognostic factors for medulloblastoma in children: conclusions from the Children's Cancer Group 921 randomized phase III study. *J Clin Oncol* **17**: 832–845

*© 2001 Cancer Research Campaign*
